# Closing the knowledge–practice gap in veterinary antimicrobial stewardship: why competency-based continuing professional development is needed in veterinary systems

**DOI:** 10.3389/fvets.2026.1825253

**Published:** 2026-05-08

**Authors:** Eugene Chisela Bwalya, Chikwanda Chileshe, Rachel Mwenda, Bruno Phiri, Kaluba Chibango, Victor Chishimba, Kenneth Chawinga, Taona Sinyawa, Edwin Sianzinda, Fusya Goma, Ntombi B. Mudenda, Chisoni Mumba

**Affiliations:** 1 School of Veterinary Medicine, University of Zambia, Lusaka, Zambia; 2 Zambia National Public Health Institute, Antimicrobial Resistance Coordinating Committee, Lusaka, Zambia; 3 Zambia Institute of Animal Health, Mazabuka, Zambia; 4 Department of Veterinary Services, Ministry of Fisheries and Livestock, Lusaka, Zambia

**Keywords:** antimicrobial resistance, antimicrobial stewardship, competency-based education, continuing professional development, veterinary paraprofessionals, veterinary workforce development, Zambia

## Abstract

Antimicrobial resistance (AMR) is a major threat to global health, livestock productivity, and food systems. Veterinary professionals play a central role in antimicrobial stewardship (AMS), yet growing evidence suggests that knowledge of AMR does not consistently translate into responsible antimicrobial use (AMU) in veterinary practice. One major limitation of existing training approaches is their emphasis on knowledge dissemination rather than the development of applied competencies required for stewardship decision-making. In this perspective, we argue that veterinary antimicrobial stewardship requires a shift from knowledge-oriented training to competency-based professional development. Drawing on experiences from Zambia's poultry sector and emerging global frameworks in competency-based veterinary education, we propose a conceptual framework illustrating how competency-based continuing professional development (CPD) can bridge the knowledge–practice gap in veterinary antimicrobial stewardship. We further present a structured competency translation model for designing stewardship-oriented CPD programs tailored to veterinary professionals working in resource-constrained livestock systems. Strengthening competencies in clinical decision-making, diagnostic stewardship, disease prevention, responsible antimicrobial prescribing, farmer communication, and regulatory compliance may represent a critical strategy for improving antimicrobial stewardship within veterinary service systems. This perspective highlights the importance of competency-based workforce development as a key component of national AMR action plans and broader One Health strategies.

## 1 The veterinary antimicrobial stewardship paradox

Antimicrobial resistance (AMR) is widely recognized as one of the most pressing global health challenges, threatening human health, livestock productivity, and food systems. Antimicrobial use (AMU) in veterinary settings contributes significantly to the selection and spread of resistant pathogens across animal, human, and environmental interfaces (1). As a result, antimicrobial stewardship (AMS) has emerged as a central pillar of global strategies aimed at mitigating AMR within the One Health framework.

Veterinary professionals, including veterinarians and veterinary paraprofessionals (VPPs), play a critical role in shaping antimicrobial use practices in livestock production systems. Their responsibilities include diagnosing animal diseases, prescribing antimicrobial treatments, advising farmers on disease prevention strategies, and ensuring compliance with veterinary drug regulations. However, despite growing awareness of AMR among veterinary professionals, inappropriate antimicrobial use continues to be reported in many livestock systems.

This situation reflects what may be described as a veterinary stewardship paradox: veterinary professionals increasingly understand the scientific basis of antimicrobial resistance, yet stewardship principles are not consistently translated into everyday clinical decision-making. In many settings, veterinary prescribing decisions occur within complex socio-economic environments characterized by limited diagnostic infrastructure, farmer expectations for rapid treatment, commercial pressures associated with livestock production, and weak regulatory enforcement (2). Under such conditions, antimicrobial use decisions are shaped not only by scientific knowledge but also by practical competencies such as clinical judgement, diagnostic reasoning, communication skills, and herd health management.

## 2 Why knowledge-based training is not enough

Continuing professional development (CPD) has long been recognized as an important mechanism for strengthening professional competencies among veterinarians throughout their careers (3, 4). Many AMR training initiatives have therefore focused on raising awareness of antimicrobial resistance and promoting responsible antimicrobial use through educational workshops and training programs.

However, traditional CPD approaches often rely heavily on lecture-based teaching and information dissemination. While such approaches can improve theoretical knowledge, they may not adequately equip veterinary professionals with the practical skills required to implement antimicrobial stewardship in real-world practice. Veterinary decision-making frequently involves complex trade-offs between animal health, economic pressures, farmer expectations, and regulatory requirements.

Consequently, improving AMR knowledge alone may be insufficient to change antimicrobial prescribing behavior. This observation is consistent with broader literature on professional education, which suggests that effective behavior change requires the development of practical competencies that integrate knowledge, skills, and professional judgement (5–7).

## 3 Competency-based education as a solution

Competency-based education (CBE) has emerged as a transformative approach in professional training systems, particularly in health professions education. Unlike traditional time-based training models, competency-based education focuses on the development and assessment of observable professional competencies that reflect real-world practice requirements (6, 8).

Within veterinary medicine, global initiatives such as Competency-Based Veterinary Education (CBVE) and the competency guidelines developed by the World Organisation for Animal Health provide structured frameworks for defining essential professional competencies across veterinary workforces (9–11). These frameworks emphasize the integration of technical knowledge with clinical reasoning, communication skills, ethical responsibility, and systems thinking.

Competency-based approaches are particularly relevant to antimicrobial stewardship because veterinary prescribing decisions are embedded within complex livestock production systems where clinical, economic, and social factors intersect (1). Strengthening the competencies required to navigate these decision environments may therefore represent a critical strategy for improving antimicrobial stewardship in veterinary practice.

## 4 Bridging the knowledge–practice gap

**Figure 1:**
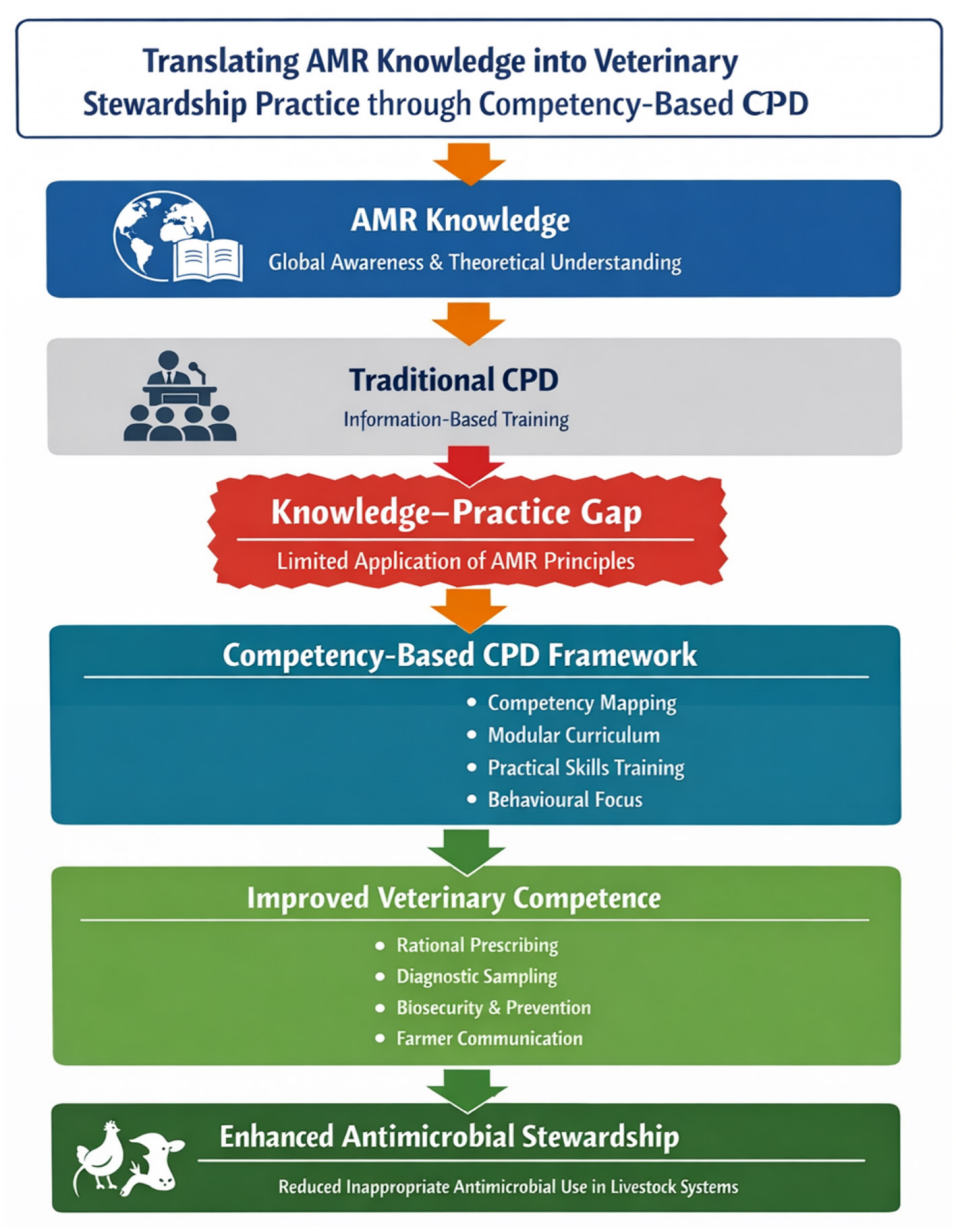
Conceptual framework (developed by the authors).

Figure 1 illustrates a conceptual framework describing how competency-based continuing professional development can bridge the gap between AMR knowledge and stewardship practice.

This framework illustrates the conceptual pathway through which competency-based professional development can transform theoretical understanding of antimicrobial resistance into practical antimicrobial stewardship behaviors within veterinary service systems. The upper section of the framework represents the current global situation in which veterinary professionals possess increasing awareness of AMR through traditional knowledge-based training and continuing professional development programs. However, such training approaches often emphasize information transmission rather than applied competencies, resulting in a persistent knowledge–practice gap in which AMR principles are not consistently implemented in day-to-day veterinary decision-making.

The central component of the framework introduces competency-based CPD as a translational mechanism designed to bridge this gap. The framework emphasizes four core elements of competency-oriented training: competency mapping, modular curriculum design, practical skills training, and behavioral learning approaches. Behavioral learning approaches are training strategies that focus on translating knowledge into observable changes in professional practice. These components enable veterinary professionals to translate theoretical stewardship principles into observable professional competencies.

The lower section of the framework illustrates the anticipated outcomes of competency-based training, including improved veterinary competence in rational antimicrobial prescribing, diagnostic sampling, disease prevention and biosecurity management, and effective farmer communication. Collectively, these competencies would strengthen antimicrobial stewardship practices within livestock production systems and contribute to the reduction of inappropriate antimicrobial use. The framework highlights the role of competency-based professional development as a critical strategy for operationalising antimicrobial stewardship within veterinary workforce development and national AMR mitigation efforts.

## 5 A competency translation model for veterinary stewardship training

To operationalise competency-based antimicrobial stewardship training, a structured curriculum development process is required. Drawing on emerging experiences from veterinary training initiatives in low- and middle-income countries, a competency translation model can be proposed for designing stewardship-oriented CPD programs. The model involves five key stages which we highlight from a case study of Zambia through a project that was funded by the International Centre for Antimicrobial Resistance (ICARS). The five stages included: formation of a multidisciplinary curriculum development team, competency mapping, curriculum translation and modular design, development of training materials and pilot implementation and evaluation.

### 5.1 Formation of the curriculum development team

A call for expressions of interest was circulated through the Veterinary Association of Zambia. Selection criteria included expertise in veterinary clinical practice, curriculum development or pedagogy, poultry health and production, antimicrobial resistance and stewardship. Applicants were also assessed on prior experience in training or capacity building and familiarity with competency-based approaches. A final team of five experts representing academia, private practice, government veterinary services, and industry was assembled. Diversity in professional experience was intentional to ensure that the curriculum reflected real-world field challenges and service delivery contexts.

### 5.2 Competency mapping

The development team conducted a structured workshop to map core competencies required for effective veterinary practice, referencing international competency frameworks and local veterinary service needs as guided by Mumba et al. (12). Competencies were grouped into domains including clinical decision-making, biosecurity and disease prevention, diagnostic sampling and evidence-based practice, prudent antimicrobial use and AMS, and farmer communication and advisory skills. Each competency was expressed as an observable behavior with measurable outcomes appropriate for CPD learners. This mapping process produced a competency framework that functioned as the backbone of the curriculum required for effective poultry health management, responsible antimicrobial use, and sustainable production practices as shown in Table 1.

**Table 1:** Competency framework of the curriculum.

Selected competency	Critical skill addressed
1. Poultry husbandry and production management	To strengthen knowledge of housing, nutrition, and welfare practices that influence flock health and productivity.
2. Disease recognition and biosecurity	To enable early identification, prevention, and control of poultry diseases, thereby reducing dependence on antimicrobials.
3. Prudent antimicrobial use and antimicrobial stewardship	To ensure veterinary paraprofessionals understand the principles of responsible drug use, withdrawal periods, and alternatives to antimicrobials.
4. Laboratory sampling and diagnostics	To enhance evidence-based decision-making and improve surveillance capacity.
5. Farmer communication and advisory skills	To empower VPPs to deliver effective farmer education, behavioral change communication, and field-based extension services.

### 5.3 Curriculum structuring and syllabus development

Each competency cluster was translated into modular course syllabi. For each module, the development team specified: competency-linked learning outcomes, detailed content outlines, suggested instructional strategies (e.g., case studies, practical demonstrations, group work), and assessment activities aligned with targeted competencies. The resulting modules were designed to be delivered independently or combined as a comprehensive CPD package. The modular design also allowed for future adaptation to different species, production systems, or thematic emphases.

### 5.4 Development of training materials

A technical writing workshop was convened to transform the syllabi into learner-centred training resources as guided by Pinto et al., (13). Three primary outputs were developed:

a) Trainee manual—outlining key concepts, practical guidance, and field-relevant examples.
b) Facilitator guide—providing session plans, learning outcomes, instructions for activities, and assessment suggestions.
c) Presentation slides—to support structured and visually guided delivery.

Adult learning principles, including experiential learning, problem-based learning, and opportunities for reflection, were integrated into exercises and assessments. Draft materials underwent internal and external peer review by veterinary educators, clinical practitioners, and AMS specialists to ensure clarity, technical accuracy, and alignment with the competency framework.

### 5.5 Pilot testing and outcomes

The participatory process resulted in a competency framework that covered technical, clinical, diagnostic, and advisory domains. Eight curriculum modules were developed, each with clearly defined competency-linked learning outcomes, content outlines, and associated training resources (trainee manuals, facilitator guides, and slides) Table 2. The modular structure enabled flexible configuration for different audiences and training durations.

**Table 2:** Modular structure learning, outcomes and duration.

Unit name	Learning outcomes	Unit duration
Unit 1: Poultry Production as a Business	• Describe different elements to consider when developing a poultry business plan • Understand poultry production cycle management • Describe essential poultry production requirements • Perform financial analysis of poultry production	2 h 10 min
Unit 2: Antimicrobial Resistance (AMR) in the Zambian Poultry Sector	• Discuss Antimicrobial Resistance and its impact • Understand AMR surveillance in Zambia • Discuss AMR in poultry in Zambia • Outline the role of VPPs in AMR mitigation and prevention	2 h 25 min
Unit 3: Common Diseases of Broilers and Layers in Zambia	• Identify common diseases affecting broilers and layers • Describe the pathological features and lesions associated with common poultry disease in Zambia • Conduct a postmortem in poultry as an aid in disease diagnosis • Apply knowledge of disease clinical signs and pathology for early detection, prevention and control strategies	4 h 45 min
Unit 4: Disease Prevention and Control in Broiler and Layer Production	• Explain the principles of disease transmission and the factors that influence disease outbreaks in poultry farms • Implement comprehensive disease prevention and control measures on poultry farms • Recognize the importance of record-keeping and monitoring for early disease detection and management	1 h 20 min
Unit 5: Application of Biosecurity Measures to Prevent Infections in Poultry Farming	• Explain the importance of biosecurity in poultry • Describe biosecurity measures applied on poultry farms • Understand biosecurity standards and guidelines	1 h 55 min
Unit 6: Sample Management for Diagnosis and Surveillance in the Poultry Sector	• Describe sample requirements for diagnosis and surveillance in poultry • Collect samples for diagnosis and surveillance • Package samples for diagnosis and surveillance • Label and document samples for transportation	1 h 50 min
Unit 7: Antibiotics and their Alternatives in Poultry	• Explain the use of antibiotics in poultry production • Describe the considerations for antibiotic use in poultry • Explain antibiotic alternatives	1 h 20 min
Unit 8: Veterinary Practice Regulation and Role of VPPs	• Understand regulatory framework governing veterinary practice • Describe registration of veterinary paraprofessionals • Understand the importance of compliance with regulations • Understand the roles of veterinary paraprofessionals in veterinary regulation	1 h 50 min
Unit 9: Enhancing Poultry Farmer Engagement and Extension Skills	• Describe the principles of Extension services • Explain the importance of Extension services • Explain key elements of effective communication	1 h 45 min

The curriculum was pilot tested with a cohort of 20 Veterinary Paraprofessionals (VPPs) from five districts. The pilot workshop was delivered over 7 days and used a mix of instructional methods, including: interactive plenary discussions, practical sessions (e.g., poultry post-mortem examinations, biosecurity assessments), competency demonstrations and role-play and communication exercises simulating farmer interactions.

Participants completed pre-training and post-training assessments constructed directly from the competency framework, focusing on knowledge and applied problem-solving. Additionally, structured evaluation forms captured participants' perceptions of content relevance, clarity, delivery methods, and applicability to their daily practice.

Post-training assessment scores 21/24 (87.5%) showed measurable improvement relative to pre-training scores 20/24 (83%), indicating enhanced understanding of poultry production, disease control, and AMS concepts. Participants demonstrated improved ability to identify risk factors for disease, propose appropriate preventive strategies, and justify antimicrobial use decisions. Participants' training evaluation (pre-training assessment) revealed that majority had challenges in understanding poultry production as a business, followed by veterinary practice regulation, and specimen management for diseases diagnosis. This observation reflects serious gaps in the undergraduate curriculums for VPPs.

## 6 Implications for AMR policy and veterinary workforce development

Strengthening veterinary antimicrobial stewardship requires more than regulatory controls or awareness campaigns. It requires investment in veterinary workforce development to ensure that veterinary professionals possess the competencies necessary to implement stewardship principles in complex livestock production systems.

Veterinary paraprofessionals play an especially important role in many LMIC veterinary systems, where they often deliver the majority of frontline animal health services (10, 14). Strengthening the competencies of this workforce through structured CPD programs may therefore represent a practical strategy for improving antimicrobial stewardship at scale.

Competency-based professional development should therefore be considered a key component of national AMR action plans and broader One Health capacity-building strategies. Integrating stewardship competencies into veterinary education and professional development programs may significantly strengthen the ability of veterinary systems to mitigate antimicrobial resistance.

## 7 Conclusion

Antimicrobial stewardship in veterinary systems cannot rely solely on improving awareness of antimicrobial resistance. Instead, stewardship requires the development of practical professional competencies that enable veterinary professionals to translate knowledge into responsible antimicrobial use in real-world practice.

Competency-based continuing professional development offers a promising pathway for bridging the knowledge–practice gap in veterinary antimicrobial stewardship. Competency-based training programs may strengthen stewardship behavior and improve antimicrobial use within livestock production systems by focusing on measurable competencies linked to veterinary practice.

Investing in competency-based veterinary workforce development should therefore be recognized as a critical component of global strategies aimed at addressing antimicrobial resistance within the One Health framework.

Future research should evaluate the long-term impact of competency-based professional development on antimicrobial prescribing behavior and antimicrobial use patterns in livestock production systems.

## Data Availability

The original contributions presented in the study are included in the article/supplementary material, further inquiries can be directed to the corresponding author.
